# Vitellogenin induction in caudal fin of guppy (*Poecilia reticulata*) as a less invasive and sensitive biomarker for environmental estrogens

**DOI:** 10.1038/s41598-017-06670-6

**Published:** 2017-08-09

**Authors:** Jun Wang, Shuwei Ma, Zhenzhong Zhang, Mingyi Zheng, Yifei Dong, Shaoguo Ru

**Affiliations:** Marine Life Science College, Ocean University of China, Qingdao, 266003 China

## Abstract

Guppy (*Poecilia reticulata*) is an ideal model for studying environmental estrogens, and its large caudal fin has a high capacity to regenerate. This study analyzed the feasibility of caudal fin for detecting vitellogenin (Vtg), the most commonly used biomarker of environmental estrogens. Firstly, a sandwich ELISA for guppy Vtg was developed using purified lipovitellin and its antibody and it had a working range of 7.8–1000 ng/mL and detection limit of 3.1 ng/mL. The ELISA was used to detect tissue distribution of Vtg. In male guppy exposed to 50 and 100 ng/L 17*β*-estradiol (E_2_), Vtg concentration in caudal fin was higher than that in whole fish, brain, eyes, gonad, and skin, and was close to that in the liver. Furthermore, male guppies were exposed to environmental concentrations of 17*a*-ethinylestradiol (EE_2_) and bisphenol S (BPS) to validate the utility of caudal fin Vtg for detecting estrogenic activities. The lowest observed effect concentration of EE_2_ and BPS were lower than 2 ng/L and 1 μg/L, which were below or equal to the values reported for other species, demonstrating that caudal fin Vtg was highly sensitive to estrogenic chemicals. Therefore, caudal fins of guppies are suggested as alternative samples for Vtg biomarker detection.

## Introduction

Environmental estrogens have aroused great concern worldwide owing to their adverse effects on wildlife health, including altered sex hormone levels^1^, gonadal abnormalities, reduced fertility^2^, and feminization of males^3^. To screen chemicals with estrogenic activity, US Environmental Protection Agency and Organization for Economic Cooperation Development (OECD) have developed test guidelines using small fish species as model organisms and vitellogenin (Vtg) as a core biomarker^4, 5^. Currently, plasma and whole-body homogenates (WBH) are normally used for Vtg detection, which involves killing experimental organisms^6, 7^. In some cases, such as field sampling of natural populations and serial monitoring of the effects of estrogenic chemicals on experimental organisms, a less invasive sampling method for repeated Vtg measurements is needed. Several studies reported that skin mucus was an alternative choice for Vtg quantification^8, 9^. However, Maltais *et al*.^10^ and our previous study^11^ found that Vtg levels in fish skin mucus were considerably lower than those in plasma, demonstrating that surface mucus Vtg induction exhibited a lower sensitivity to estrogenic chemicals. Recently, Zhong *et al*.^12^ reported that Vtg could be detected in various extrahepatic tissues of 17*α*-ethinylestradiol (EE_2_)-exposed male zebrafish (*Danio rerio*), and recommended skin and eye tissues as for Vtg analysis. Sampling of these tissues would kill the fish, thus it was unable to measure Vtg induction at a later point in time. By contrast, caudal fin has a high capacity to regenerate, and zebrafish caudal fin could quickly regenerate in approximately two weeks after 95% excision^13^. We speculated that using caudal fins as the sampling tissue could be helpful for continuous measurement of Vtg biomarker. However, it is still unclear whether caudal fin contains Vtg and whether the sensitivity of Vtg induction in caudal fin is high enough to detect weak estrogenic activity.

**Figure 1:**
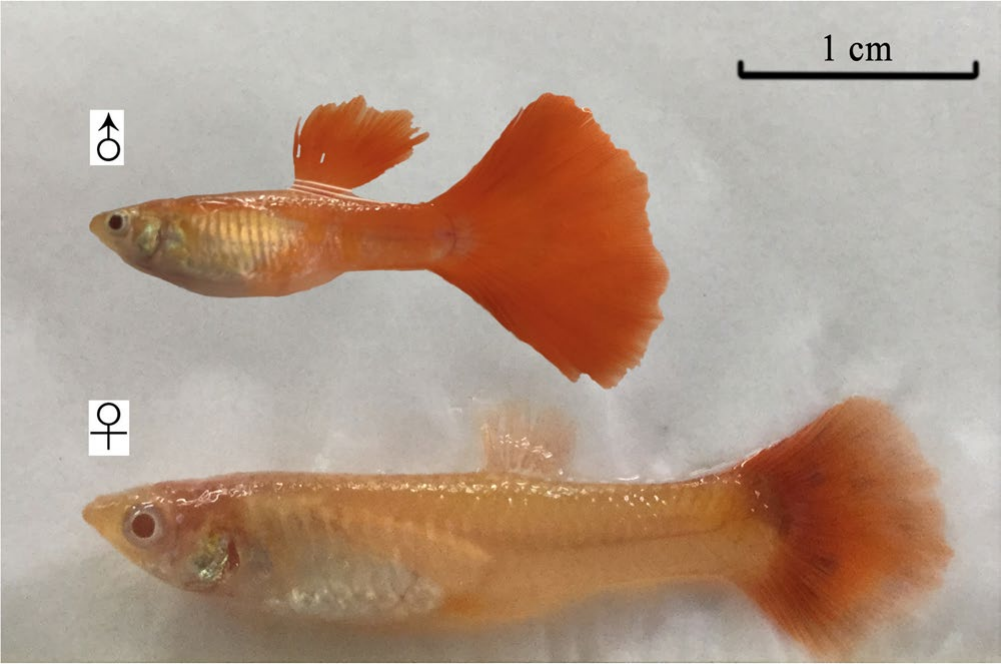
Photograph of male and female guppy (*Poecilia reticulata*).

The guppy (*Poecilia reticulata*) has a large caudal fin, which is almost half of their total length and can regenerate quickly and reliably after amputation, restoring both size and shape^14, 15^. Thus, it provides a good model to evaluate the feasibility of caudal fin Vtg as a less invasive biomarker for detecting environmental estrogens. Guppy is easily cultured in the laboratory and easy to identify gender due to its obvious secondary sexual characteristics (Fig. 1). Moreover, it was reported that guppy was more sensitive to exogenous estrogens than zebrafish and rainbow trout^16, 17^. Guppy was therefore considered an ideal model to study environmental estrogens^18, 19^. However, Vtg in guppy is still detected using qualitative methods, such as SDS-PAGE and Western blot analysis, and its quantitative tool has not been developed owing to the lack of anti-guppy Vtg antibody until now^17, 20^.

In the present study, guppy lipovitellin (Lv), the main egg yolk protein derived from Vtg, was purified and used to prepare antibodies. Using the purified Lv and its antibody, a sandwich ELISA for measuring guppy Vtg was established. Subsequently, Vtg induction in the WBH, caudal fin, liver, brain, eye, gonad, and skin tissues of male guppy exposed to different concentrations of 17*β*-estradiol (E_2_) were measured by Western blot and the sandwich ELISA to evaluate the possibility of using caudal fin for Vtg detection. Furthermore, the reliability of Vtg in caudal fin as a biomarker of estrogenic contamination was validated by quantifying Vtg levels in WBH, liver, and caudal fin of male guppy exposed to environmental concentrations of two different exogenous estrogens, EE_2_ and bisphenol S (BPS), which are two commonly detected estrogenic chemicals in aquatic environments.

## Results

### Purification of Lipovitellin

**Figure 2:**
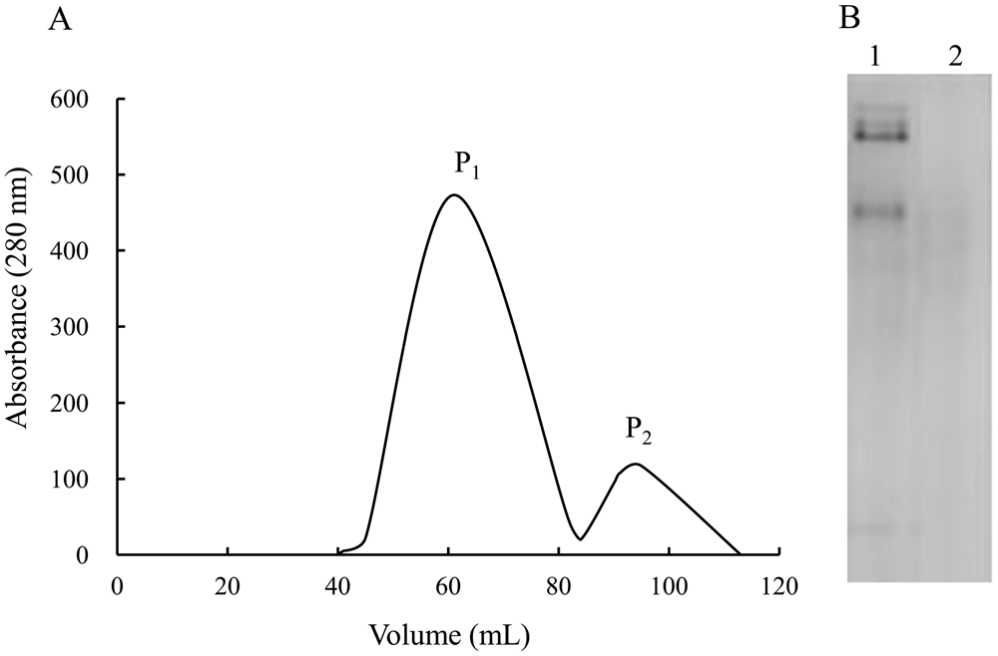
Elution profiles of ovarian homogenates on a Sephacryl S-300 column (**A**) and Native-PAGE (4–7.5%) detection of peak elution (**B**). lane 1, Peak P_1_; lane 2, Peak P_2_.

**Figure 3:**
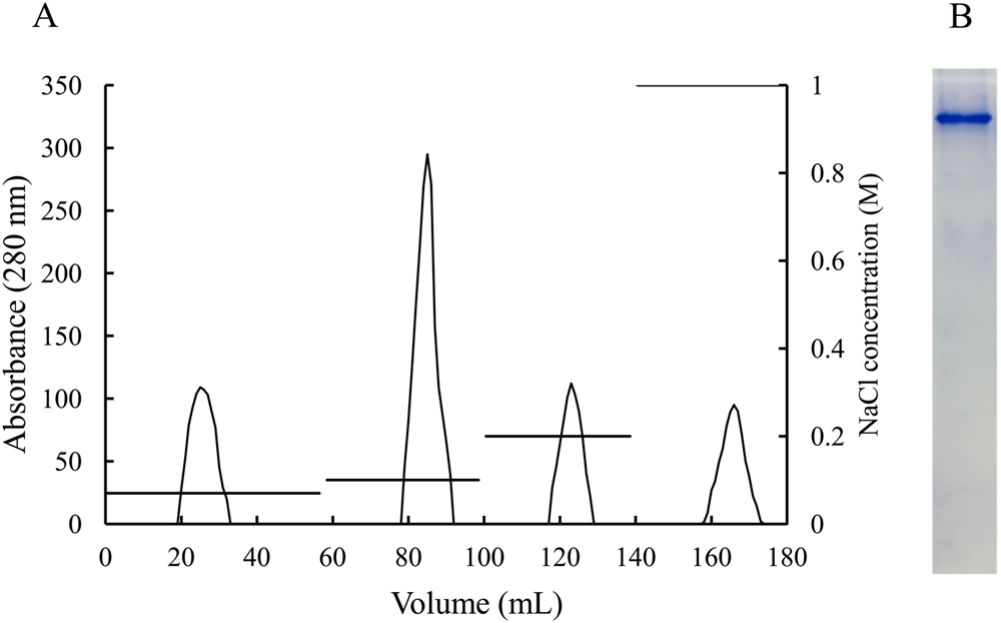
DEAE anion exchange column of guppy Lv (**A**) and Native-PAGE (4–7.5%) of the peak eluted with buffer containing 0.1 M NaCl (**B**).

Ovarian homogenate yielded two peaks in gel filtration chromatography and the first peak appeared clear protein bands (Fig. 2). Thus fractions of this peak were collected and subjected to anion exchange chromatography. The main peak was found in 25 mM Tris-HCl buffer containing 0.1 M NaCl and it showed a single band in Native-PAGE (Fig. 3).

### Characterization of Lipovitellin

**Figure 4:**
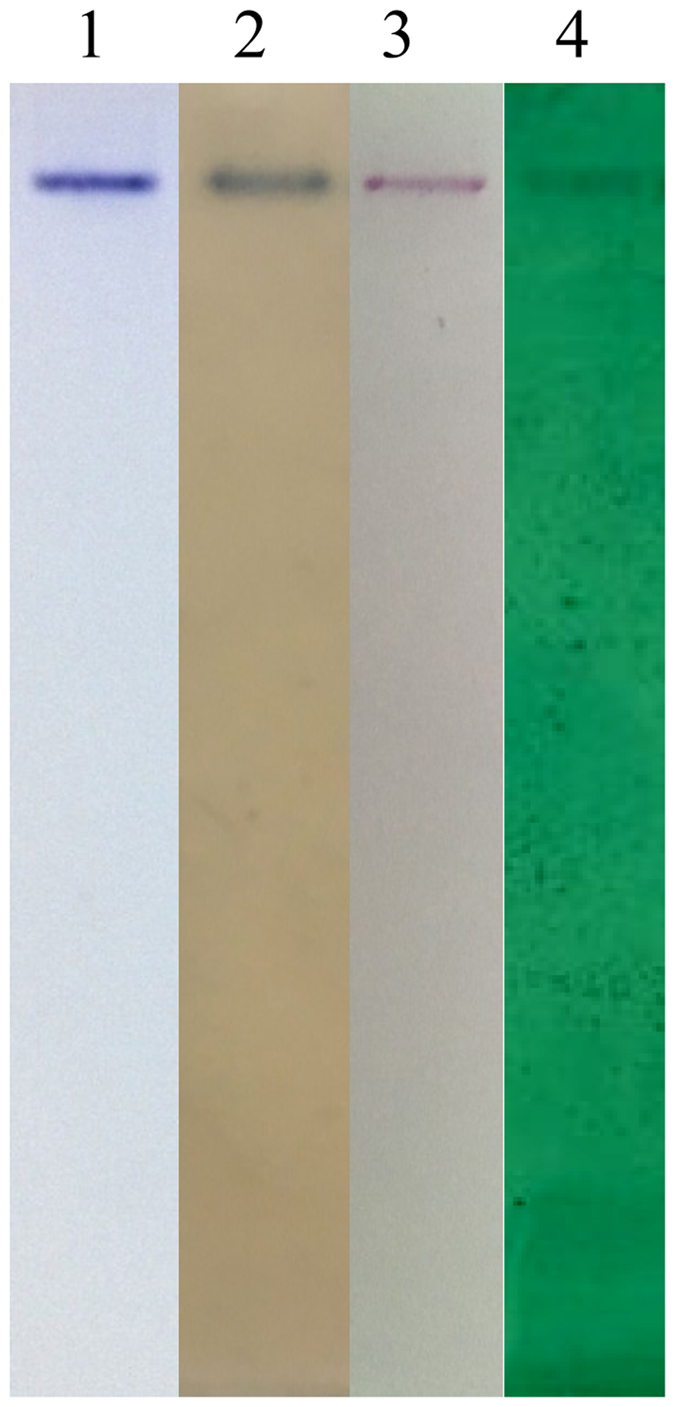
Specific staining of carbohydrate (lane 2), phosphorus (lane 3) and lipid components (lane 4) for the purified protein. Lane 1 was stained with CBB.

The purified protein was stained positively with Schiff reagent, methyl green, and Sudan black B (Fig. 4), confirming that it was a phospholipoglycoprotein.

**Figure 5:**
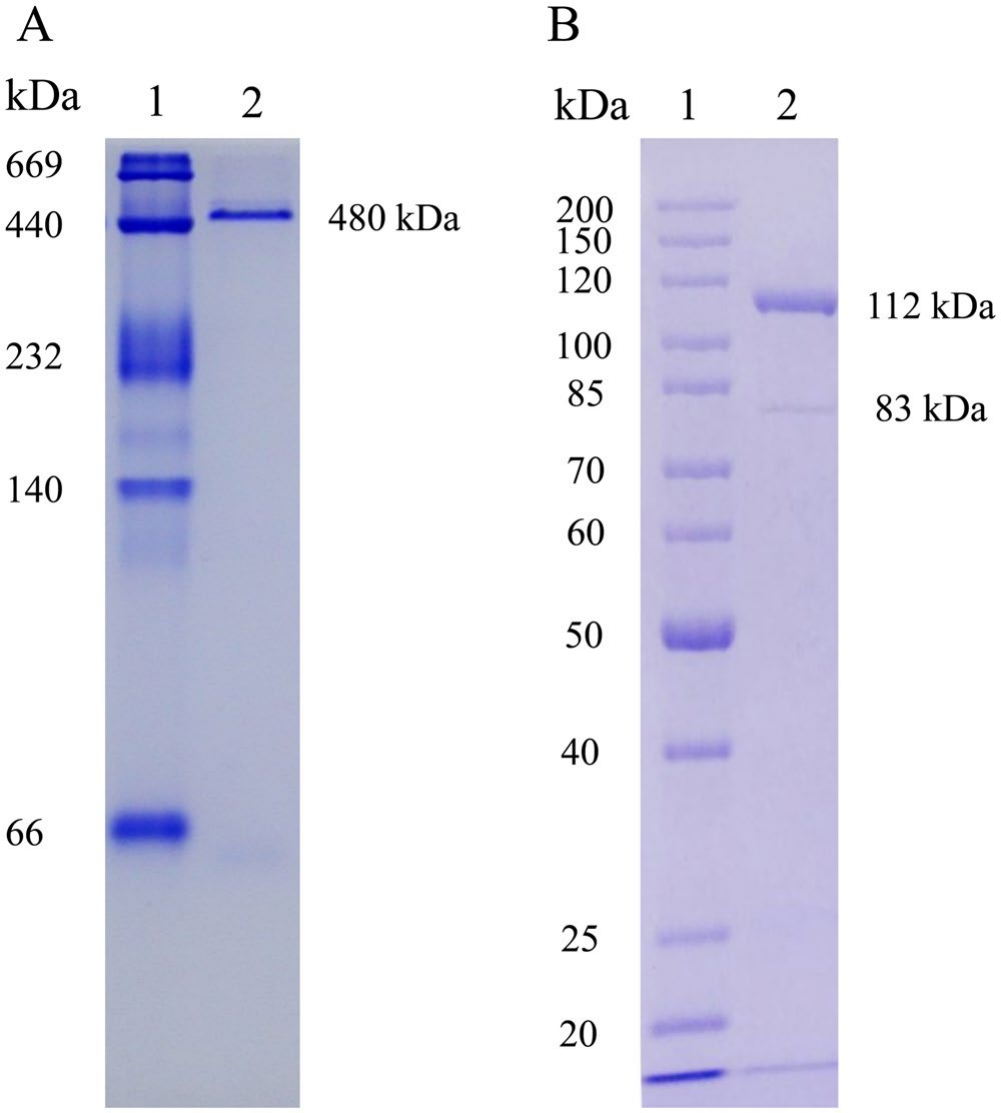
Native PAGE (4–7.5%) (**A**) and SDS-PAGE (**B**) electrophoretic patterns of purified guppy Lv. lane 1, protein maker; lane 2, the purified Lv.

The apparent molecular weight of guppy Lv was estimated to be approximately 480 kDa by native-PAGE (Fig. 5A). On SDS-PAGE, guppy Lv resolved into a major band at 112 kDa and a minor band at 83 kDa (Fig. 5B).

### Specificity of the antiserum to vitellogenin

**Figure 6:**
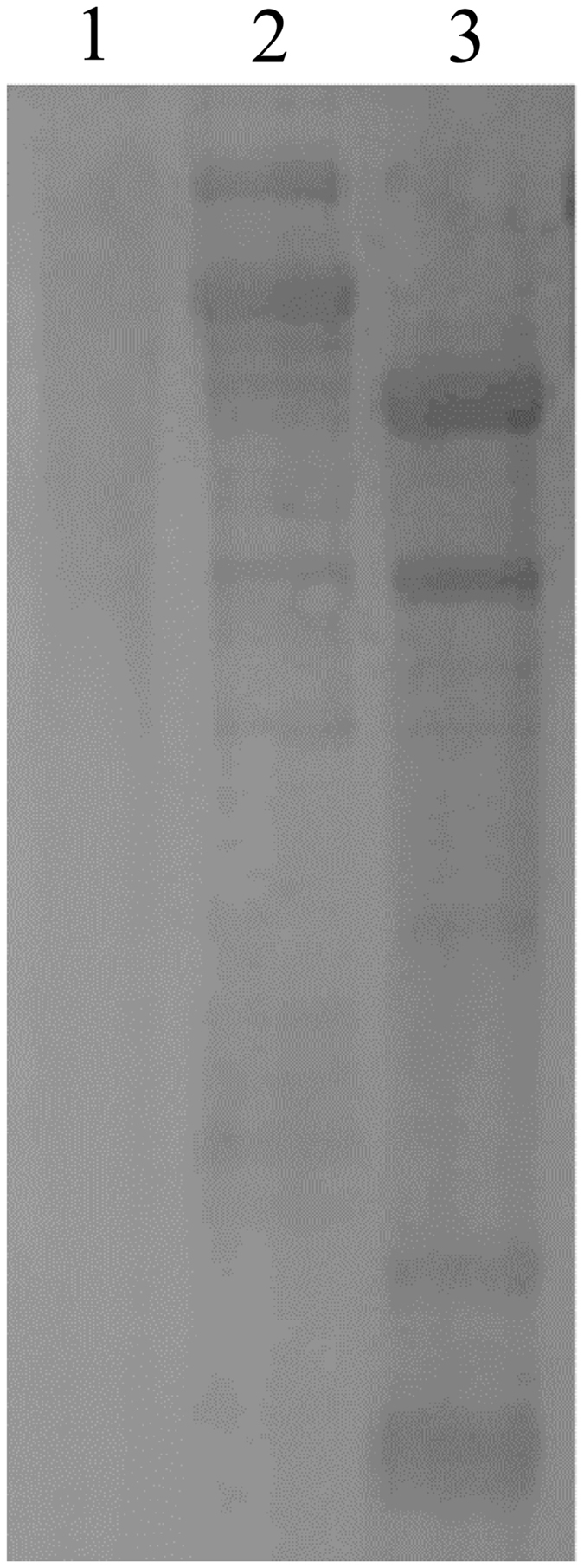
Western blot analysis of WBH from male (lane 1), E_2_-exposed male guppy (lane 2), and purified Lv (lane 3) using anti-Lv antiserum as primary antibody.

Western blot results showed that anti-Lv antibody detected several bands in WBH from E_2_-exposed male guppy and the purified Lv, while no visible bands were detected in WBH from male fish (Fig. 6).

### Development and validation of an ELISA for guppy vitellogenin

**Figure 7:**
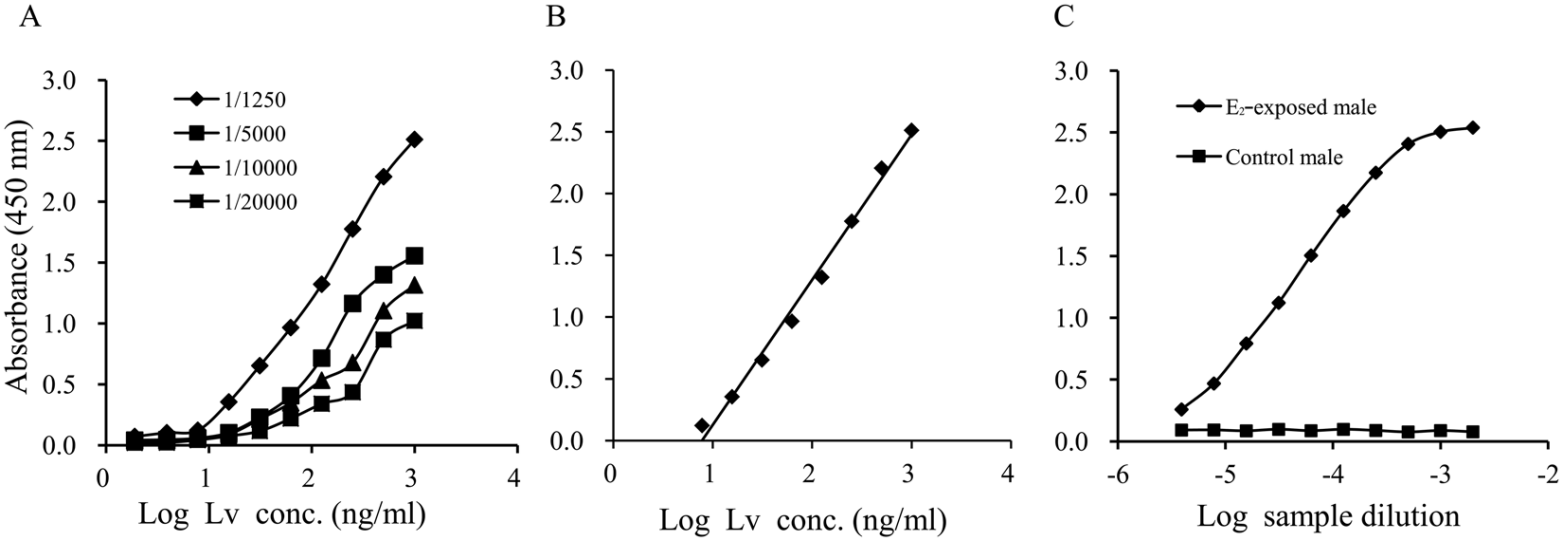
Determination of optimal dilution of HRP-labeled anti-Lv IgG (**A**), and a representative standard curve obtained for guppy Lv (**B**) and WBH dilution curves of control male and E_2_-exposed male guppy in sandwich ELISA (**C**).

Serial dilutions of HRP-labeled anti-Lv antibody were used as detecting antibodies to determine the optimal condition of the assay (Fig. 7A). When the detecting antibody was diluted 1:1250 times, the curve showed a wide linear work range with high maximum absorbance of about 2.6. Under this condition, the working range of this assay was 7.8–1000 ng/mL (y = 1.1747x − 1.0473, *R* ^2^ = 0.992), and the detection limit was estimated as 3.1 ng/mL (Fig. 7B). Moreover, the Lv standard curve was parallel to dilutions of WBH from E_2_-exposed male guppy over the entire working range of the assay, while control male WBH showed no reaction in the ELISA (Fig. 7C). The matrix effects for caudal fin and WBH were found to be reduced to acceptable levels when they were diluted 20-fold and 40-fold, respectively (Figs S1 and S2). At these levels of dilution, the matrix interferences were similar to those observed in the case of the matrix free buffer (PBST). Therefore, the practical detection limit for Vtg in caudal fin and WBH samples were 62 and 124 ng/mL, respectively.

The intra- and inter-assay CVs of the ELISA within the working range were 0.73~3.85% and 0.36~7.43%, respectively (Tables 1 and 2).

**Table 1:** Precision tests of the sandwich ELISA.

Variation	g-Lv concentration *(*ng*/*ml*)*	N	CV (%)
Intra-assay	49.88 ± 1.92	8	3.85
	100.90 ± 2.26	8	2.24
	406.21 ± 6.21	8	1.53
	798.93 ± 5.87	8	0.73
Inter-assay	50.45 ± 3.08	8	6.10
	97.07 ± 7.22	8	7.43
	391.46 ± 9.81	8	2.51
	801.33 ± 2.86	8	0.36

Values of Lv concentration are expressed as mean ± standard deviation; N is the number of determinations; and CV is the coefficient of variation.

**Table 2:** Lowest observed effect concentration (LOEC) of Vtg induction in different fish exposed to waterborne 17*a*-ethynylestradiol.

Test species	Status	Exposure type and concentrations	Detected sample	LOEC Vtg indution	References
Zebrafish (*Danio rerio*)	adult male	8-day flow-through exopsure to 0.7, 2.2, 3.6, 6.6, 10.1, 13.5, 17.2, 26.1, and 90.1 ng/L	whole-body homogenate	3.6 ng/L	47
	adult male	21-day semi-static exposure to 5, 10, 25 and 50 ng/L	plasma	10 ng/L	38
	adult male	21-day semistatic exposure to 0–25 ng/L	plasma	10 ng/L	48
	juvenile	38-day semistatic exposure to 10 and 100 ng/L	whole-body homogenate	10 ng/L	49
	adult male	21-day semi-static exposure to 1.67, 3.0, 7.5, 10, and 20 ng/L	plasma	1.67 ng/L	50
	juvenile	90-day static exposure to 0.1, 1, 10, and 25 ng/L	whole-body homogenat	10 ng/L	51
Medaka (*Oryzias latipes*)	adult male	28-day static exposure to 10 and 100 ng/L	plasma	10 ng/L	52
	adult male	21 day flow-through exposure to 31.3, 62.5, 125, 250 and 500 ng/L	liver	62.5 ng/L	39
	adult male	21-day flow-through exposure to 6.2, 12.2, 24.5, 49.9, and 93.2 ng/L	plasma	24.5 ng/L	53
	adult male	14-day static exposure to 0.2, 5, 500, and 2,000 ng/L	plasma	500 ng/L	54
	juvenile	38-day semistatic exposure to 10 and 100 ng/L	whole-body homogenate	100 ng/L	49
Fathead minnows (*Pimephales promelas*)	adult male	7-day flow-through exposure to 0.5, 1, 5, 10, 50, and 100 ng/L	plasma	5 ng/L	55
	adult male	8 day flow-through exposure to 10 and 100 ng/L	plasma	10 ng/L	56
	adult male	21-day static exposure to 10, 20, and 40 ng/L	plasma	10 ng/L	40
	adult male	21-day flow-through exposure to 0.1, 1, 3, 10, and 100 ng/L	plasma	1 ng/L	41
	juvenile	21-day flow-through exposure to 2, 5, and 20 ng/L	whole-body homogenate	5 ng/L	57
Three-spined stickleback (*Gasterosteus aculeatus*)	adult male	21-day flow-through exposure to 5, 50, and 200 ng/L	plasma	50 ng/L	42
Carp (*Cyprinus carpio*)	juvenile	10-day exposure to 1, 10, 25, and 50 ng/L	plasma	10 ng/L	58
Rainbow trout (*Oncorhynchus mykiss*)	juvenile	21-day semistatic exposure to 5, 10, and 25 ng/L	plasma	5 ng/L	59
Sheepshead minnow (*Cyprinodon variegatus*)	adult male	16-day flow-through exposure to 20, 100, 200, 500, and 1000 ng/L	plasma	100 ng/L	43
Murray rainbowfish (*Melanotaenia fluviatilis*)	adult male	7-day semistatic exposure to 1, 5, 10, 50, and 100 ng/L	Plasma	5 ng/L	60
Atlantic salmon (*Salmo salar*)	juvenile	7-day static exposure to 5 and 50 ng/L	plasma	50 ng/L	61
Mummichog (*Fundulus heteroclitus*)	adult male	21-day static exposure to 1, 10, and 100 ng/L	plasma	100 ng/L	44
Guppies (*Poecilia reticulata*)	adult male	21-day semistatic exposure to 2, 10, and 50 ng/L	tail fin	2 ng/L	This study

### Distribution of Vitellogenin in male guppy exposed to E_2_

**Figure 8:**
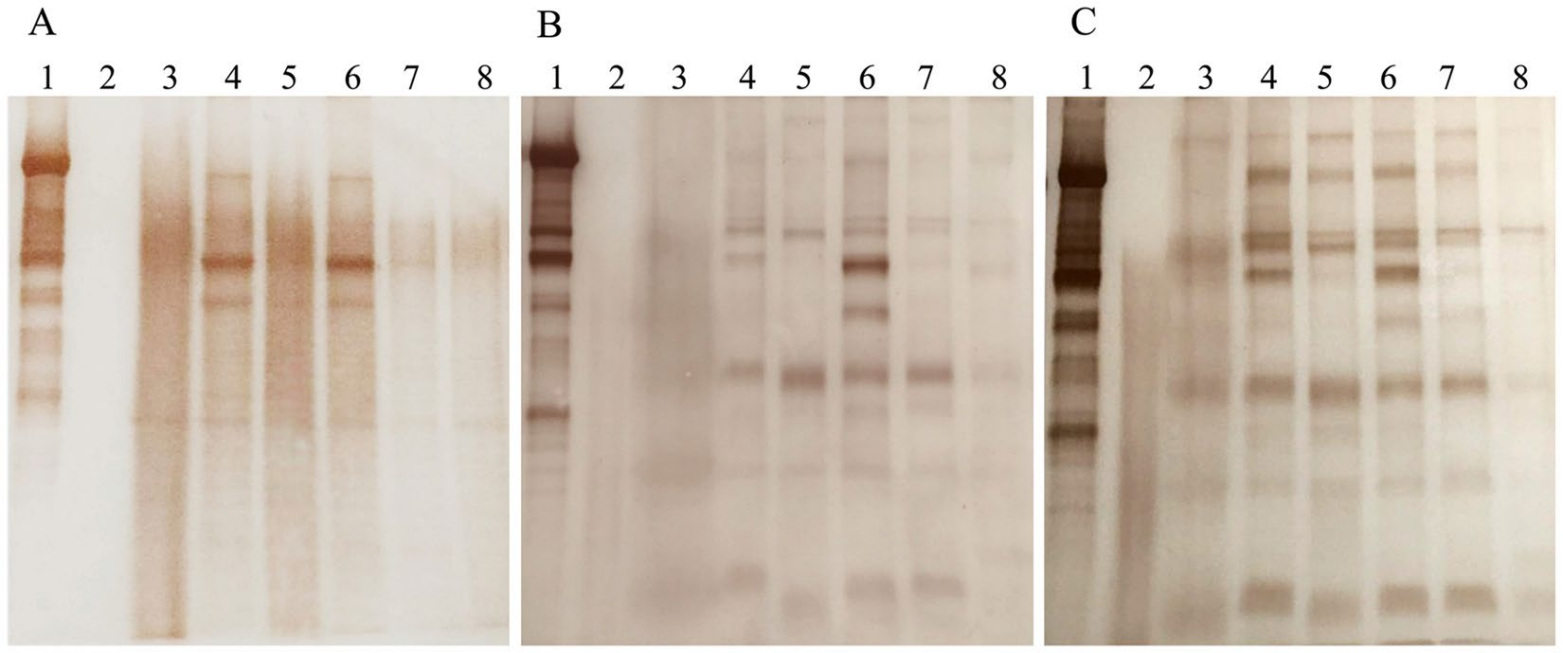
Western blot analysis of Vtg induction in different tissues of adult male guppy exposed to 50 (**A**), 100 (**B**), and 200 ng/L E_2_ (**C**) for 21 days. Lane 1, WBH of ovariectomized female fish; lane 2, WBH of control male fish; lane 3, skin; lane 4, caudal fin; lane 5, gonad; lane 6, liver; lane 7, eye; lane 8, brain.

After a 21-day exposure, the distribution of Vtg in male guppy was qualitatively detected by Western blot analysis. Prior to E_2_ exposure, there was no positive signal for Vtg in WBH of control male fish, while strong signal was found in WBH of ovariectomized female fish. After exposure to 50 ng/L E_2_ for 21 days, three clear positive bands were detected in the liver and caudal fin, which had the similar position to that of WBH from the female guppy (Fig. 8A). In 100 ng/L E_2_ exposure group, male fish showed detectable Vtg signals in skin, caudal fin, gonad, liver, eye, and brain tissues, and the strongest signal was detected in the liver (Fig. 8B). Positive bands were observed in all tissues of male fish exposed to 100 ng/L E_2_ and the number of bands was more than the fish exposed to 50 and 100 ng/L E_2_ (Fig. 8C).

**Figure 9:**
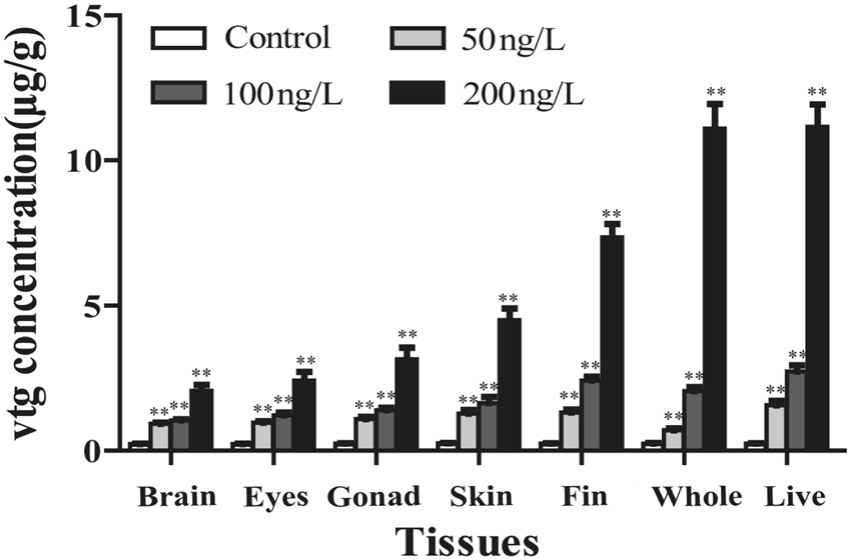
Concentrations of Vtg in guppy exposed to 50, 100, and 200 ng/L E_2_ for 21 days. Values are means ± S.D and asterisks indicate statistically significant difference from the control group (**P < 0.01).

Vtg concentrations in each tissue and WBH of male guppy in control and exposure groups were quantified by ELISA (Fig. 9). In control male guppies, Vtg values were all below the detection limit of ELISA, while concentration-dependent increases of Vtg concentrations were observed in all tissues of male guppies after 21 day exposure to E_2_. In 200 ng/L E_2_-exposed male fish, Vtg concentrations in brain, eye, testis, skin, caudal fin, whole body, and liver tissues showed an increasing tendency, and concentrations of Vtg in caudal fin, whole body, and liver were above 7 μg/g. Similarly, Vtg concentrations in caudal fin, whole fish, and liver of male fish exposed to 100 ng/L E_2_ were 2.42 ± 0.13 μg/g, 2.05 ± 0.14 μg/g, 2.73 ± 0.20 μg/g, significantly higher than other tissues in the same group (*P* < 0.01). For 50 ng/L E_2_ group, Vtg concentrations in caudal fin, whole fish and liver were 1.31 ± 0.10 μg/g, 0.71 ± 0.06 μg/g, and 1.56 ± 0.15 μg/g. Moreover, Vtg concentration in caudal fin was significantly higher than in the whole fish (*P* < 0.05).

### Caudal fin vitellogenin induction in male guppy exposed to environmental concentrations of EE_2_ and BPS

**Figure 10:**
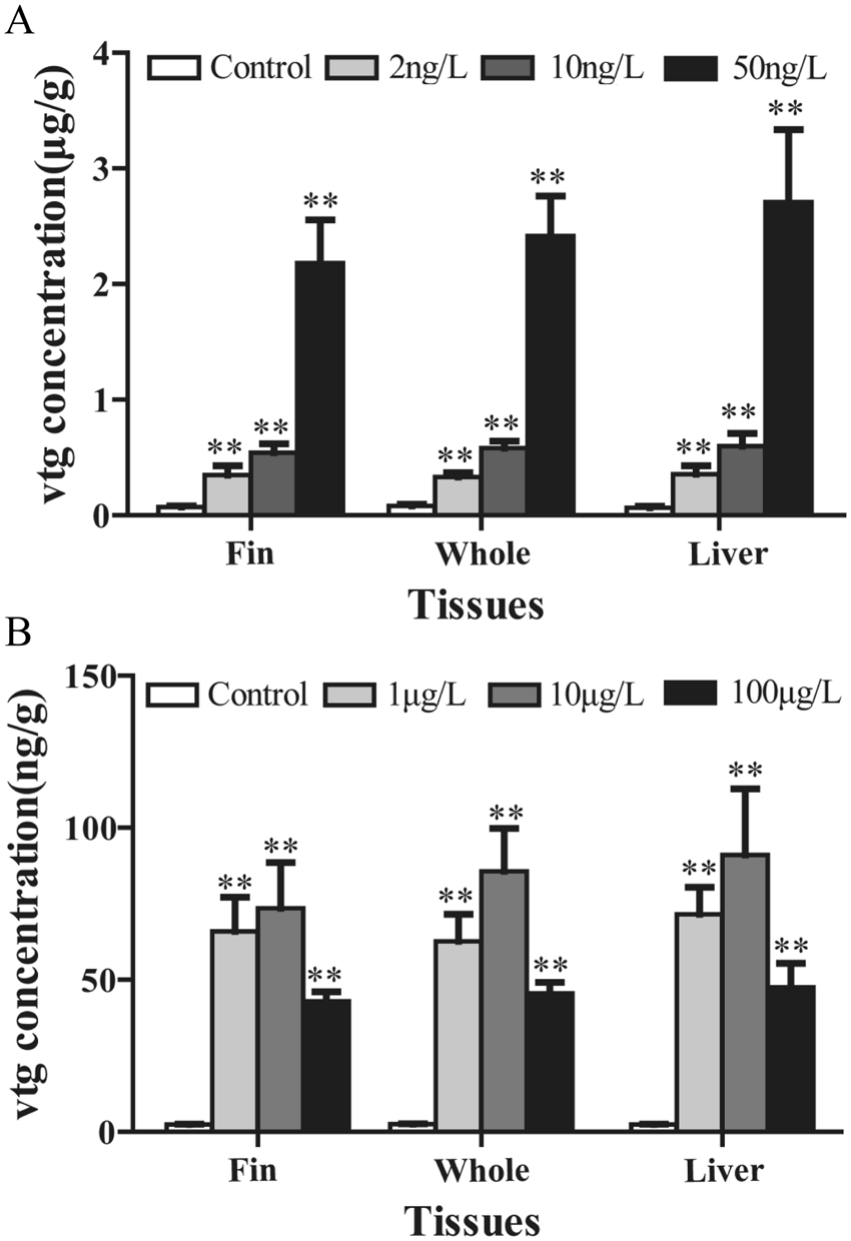
Concentrations of Vtg in guppy exposed to 2, 10, and 50 ng/L EE_2_ (**A**) and 1, 10, and 100 μg/L BPS (**B**) for 21 days. Values are means ± S.D. and asterisks indicate statistically significant difference from the control group (**P < 0.01).

Vtg concentrations in caudal fin of male guppy exposed to 2, 10, and 50 ng/L EE_2_ for 21 days were 0.35 ± 0.08 μg/g, 0.55 ± 0.08 μg/g, and 2.18 ± 0.37 μg/g, respectively (*P* < 0.01, Fig. 10A). In 2 and 10 ng/L EE_2_ exposure groups, there was no difference in Vtg concentrations between these sampled tissues. Exposure to 1, 10, and 100 μg/L BPS also increased the concentration of caudal fin Vtg significantly compared to solvent control group (*P* < 0.01, Fig. 10B). Furthermore, 10 μg/L BPS exposure induced the highest caudal fin Vtg concentration, while 100 μg/L BPS dose resulted in less induction of Vtg, which showed the same trend as Vtg in the liver and whole body.

## Discussion

In the present study, Vtg in caudal fin of guppy was found to be highly sensitive to exogenous estrogens, and it could be used as a less invasive biomarker for measuring estrogenic activity. As the most commonly used biomarker for environmental estrogens, Vtg was normally quantified by ELISA^21, 22^. Our previous study confirmed that Lv was very stable and showed the same binding efficiency to anti-Lv antibody as did Vtg. In addition, the ELISA developed using Lv as the antigen could quantify Vtg more accurately^23^. Therefore, in the present study, we chose Lv to develop the ELISA for quantification of guppy Vtg. Guppy Lv purified by a two-step chromatographic method was characterized as a phospholipoglycoprotein with an apparent molecular weight of approximately 480 kDa and produced a major band of approximately 112 kDa in SDS-PAGE, which was similar to that in other teleosts^11, 24^. Western blot elucidated that the polyclonal antibody raised against purified Lv reacted with WBH from E_2_-exposed male guppy, whereas no positive reaction occurred in control male WBH, indicating that the anti-Lv antibody was highly specific to guppy Vtg^25^. The sandwich ELISA developed using purified Lv and anti-Lv antibody had a working range of 7.8–1000 ng/mL and a detection limit of 3.1 ng/mL, which was consistent with the vaules reported for Vtg ELISA of tilapia (*Sarotherodon melanotheron*)^26^ and rare minnow (*Gobiocypris rarus*)^27^. In order to avoid the matrix effect, all tissues except caudal fin were diluted at least 1:40 in the routine assay. The average intra-assay and inter-assay CVs of Lv-based sandwich ELISA were 2.1% and 4.1%, respectively, which were lower than those of ELISAs developed using Vtg as antigen^28, 29^, demonstrating that the established ELISA had high precision. Moreover, the parallelism observed between the Lv standard curve and WBH dilution curves of E_2_-exposed male guppy demonstrated that anti-Lv antibody recognized Lv and Vtg similarly. In addition, the ELISA did not detect positive signal in control male WBH, indicating that this assay was very specific to Vtg^30, 31^. The above results confirmed that the sandwich ELISA established in the present study could accurately quantify guppy Vtg.

Guppy is a new promising model organism for studying environmental estrogens^32, 33^. Male guppies have a large caudal fin that is easily regenerated^15^. If the sensitivity of Vtg induction in caudal fin is close to that in WBH, caudal fin could replace the whole fish as the preferred sample for Vtg detection, which will be more suitable for field investigations of wild populations and for repeated vtg measurements. In the present study, Vtg concentrations in caudal fin, liver, brain, eyes, gonads, skin of male guppy exposed to E_2_ were quantified using the developed sandwich ELISA developed. It was found that exposure to 50, 100, and 200 ng/L E_2_ significantly increased Vtg concentrations in all tissues. The amount of Vtg in all exposure groups was greatest in the liver tissue, which was consistent with the tissue distribution of Vtg reported in zebrafish^12^. In the 100 ng/L E_2_-exposed group, the concentration of Vtg in caudal fin was significantly higher than that in eyes, skin, other tissues, and the whole fish. Vtg concentration in caudal fin of male fish exposed to 50 ng/L E_2_ was 1.85 times that of whole fish, and it was much closer to Vtg concentration in the liver than that in the 100 ng/L E_2_-exposed group. Evidently, Vtg induction in caudal fin had equal or even higher sensitivity than Vtg in whole fish at lower E_2_ concentrations (<100 ng/L). Owing to the ease of sampling and lower damage to fish, caudal fins can be good alternatives to WBH for the detection of Vtg in guppy.

To further test the reliability of caudal fin Vtg for the detection of estrogenic activity, the present study used two common exogenous estrogens to carry out exposure experiments. EE_2_ is the most detected synthetic estrogen that is not broken down in surface and sewage waters. Concentrations of EE_2_ in surface water of the UK, Japan, and Canada usually ranged from 1 to 20 ng/L^34, 35, 36^, although a maximum of 273 ng/L was found in US streams^37^. Many studies have reported that Vtg induction in various fish species after waterborne exposure to EE_2_ (Fig. 2). The lowest observed effect concentration (LOEC) of Vtg induction was normally 5–20 ng/L for zebrafish, medaka (*Oryzias latipes*), and fathead minnow (*Pimephales promelas*)^38, 39, 40^, except one study reported the LOEC of Vtg induction in male fathead minnow for EE_2_ was 1 ng/L^41^. For three-spined stickleback (*Gasterosteus aculeatus*), Sheepshead minnow (*Cyprinodon variegatus*), and Mummichog (*Fundulus heteroclitus*), the LOEC of Vtg induction for EE_2_ was higher than 50 ng/L^42, 43, 44^. In the present study, we found that 2 ng/L EE_2_ exposure significantly induced caudal fin Vtg, indicating that the LOEC of Vtg induction in guppy caudal fin was lower than 2 ng/L EE_2_, which was lower than the values reported for most tested species. Moreover, Vtg concentration in caudal fin was not significantly different from that in the liver and whole fish. Thus, the results demonstrated that Vtg in caudal fin of male guppy could detect weak estrogenic activity of EE_2_. In addition, this study tested the sensitivity of guppy caudal fin Vtg to BPS, which has emerged as a potential bisphenol A replacement and has been frequently detected in aquatic environments of various countries in recent years. A maximum of 7.2 μg/L of BPS was found in Adyar River of India^45^. Naderi *et al*.^46^ reported that 10 μg/L BPS exposure could significantly increase the plasma Vtg concentration in male zebrafish. In this study, 1 μg/L BPS exposure for 21 days was found to significantly increase Vtg concentration in caudal fin, indicating that caudal fin Vtg in male guppy had an equal or even higher sensitivity to BPS than plasma Vtg in zebrafish. The above results confirmed that Vtg induction in caudal fin of guppy was highly sensitive to estrogenic chemicals and could be used as a reliable biomarker for the detection of estrogenic activity in the aquatic environment, though the stress of handling fish repeatedly should then be taken into account.

In summary, a sandwich ELISA for accurate quantification of guppy Vtg was established using purified Lv and its polyclonal antibody. Subsequently, this ELISA was used to detect tissue distribution of Vtg in male guppy exposed to exogenous estrogens. Caudal fin produced Vtg in response to low concentrations of exogenous estrogens, and its Vtg concentration was significantly higher than that in the whole fish at low E_2_ concentration. Further, we found that caudal fin Vtg of guppy was capable of detecting estrogenic activities of EE_2_ and BPS at environmentally relevant concentrations. Therefore, we suggest guppy caudal fin with a high capacity to regenerate as a potential alternative sample for repeated Vtg analysis.

## Methods

### Experimental fish

Sexually mature red albino guppies (*Poecilia reticulata*) (wet mass, 0.32 ± 0.10 g; standard length, 2.2 ± 0.4 cm) were mainatined in 50-L aquaria filled with 30-L dechlorinated tap water at 26 ± 1 °C, with 7.0 ± 0.1 mg/L dissolved oxygen and 14 h:10 h light-dark cycle. The fish were fed with newly hatched brine shrimp twice and half of the aquaria water was replaced daily. In addition, the fish were handled according to the National Institute of Health Guidelines for the handling and care of experimental animals and the animal utilization protocol was approved by the Institutional Animal Care and Use Committee of the Ocean University of China.

### Lipovitellin purification

Ovaries were removed from adult female guppies and homogenized in ice-cold homogenate buffer (20 mM Tris-HCl, pH 7.5, containing 100 mM NaCl and 10 mM EDTA) using a glass homogenizer. After centrifugation at 8,000 × *g* for 10 min at 4 °C. Lv was purified from ovarian homogenates by gel filtration (Sephacryl S-300 HR 16/60 column; GE Healthcare, Uppsala, Sweden) and anion-exchange chromatography (DEAE-Sepharose FF 12/20 column; GE Healthcare, Uppsala, Sweden) according to our previously reported methods^62^. After determination of protein concentration by the Bradford assay using bovine serum albumin (BSA) as the standard, the purified Lv was stored at −80 °C.

### Lipovitellin characterization

The purified protein was analyzed using native polyacrylamide gel electrophoresis (PAGE; 4–7.5%), and the gels were stained with Coomassie Brilliant Blue (CBB), Schiff reagent, methyl green, and Sudan black B following the methods as describe by Pan *et al*.^63^.

The molecular mass of purified Lv was estimated by HMW native marker Kit (GE Healthcare, USA) according to the method described by Sun and Zhang^64^. Polypeptide components of Lv were analyzed by sodium dodecyl sulfate (SDS)-PAGE. After electrophoresis, gels were stained with CBB, and molecular weight of polypeptide units was estimated using an unstained protein ladder (20–200 kDa, Thermo Scientific, Waltham, MA, USA).

### Antibody production and label

Polyclonal antiserum against guppy Lv was raised in rabbits following routine methods. Briefly, rabbits were injected subcutaneously with 1 mL of Lv solution (800 μg) emulsified in complete Freund’s adjuvant followed by another three boosts of Lv solution (500 μg) in incomplete Freund’s adjuvant at 2-week intervals. Blood was collected, centrifuged, and anti-Lv immunoglobulin G (IgG) was purified from the supernatant by affinity chromatography on a HiTrap Protein G column (GE Healthcare). The purified IgG was labeled with horseradish peroxidase (HRP, Sigma, USA) by an improved sodium periodate-oxidation method^25^.

### Western blot of Vitellogenin

Western blot analysis was performed to check the specificity of anti-Lv antibody to guppy Vtg. The WBH of control male and E_2_-exposed male guppy, and the purified Lv were electrophoresed on SDS-PAGE and then transferred onto polyvinylidene difluoride membranes. After incubation with anti-Lv antibody at a 1:1000 dilution, the membranes were incubated with HRP-conjugated goat anti-rabbit IgG (Solarbio, Beijing, China) at a 1:2000 dilution. Finally, the membranes were visualized with freshly prepared DAB substrate.

### Sandwich ELISA

A sandwich ELISA was developed by the procedure of Mitsui *et al*.^65^ with minor modification. Microtiter plates (Costar, Cambridge, MA) were coated with 100 μL of anti-Lv IgG diluted in 0.05 M sodium carbonate overnight at 4 °C and washed three time with 200 μL/well of PBST (10 mM PBS containing 0.05% Tween-20). The wells were then incubated with 200 μL PBST containing 1% BSA for 1 h at 37 °C. After three washes with PBST, 100 μL of samples and standard serially diluted with PBST were added to each well and incubated at 37 °C for 1 h. The wells were washed five times and received 100 μL/well of HRP-labeled anti-Lv antibody at serial dilutions (1:1250, 1:2500, 1:5000, 1:10 000). After incubation at 37 °C for 1 h, the color was developed with 100 μL of tetramethylbenzidine enzyme substrate (Solarbio, China) and stopped by adding 100 μL of 2 M sulfuric acid. Absorbance values were measured at 450 nm in a plate reader. Standards and samples were run in duplicate.

The reliability of ELISA assay were evaluated by measuring its precision, sensitivity, and specificity^30^. Briefly, precision was evaluated using various purified Lv concentrations by measuring the intra- and inter-assay coefficients of variation (CV%), which were defined as the standard deviation devided by the mean and mutiplied by 100. The specificity was assessed by comparing curves of serial dilutions of WBH from E_2_-treated male guppy and the Lv standard curve. The limit of detection was defined as the concentration corresponding to the mean of the absorbance values for 12 replicates of the zero standards plus two times the standard deviation. Additionally, the matrix effect of caudal fin and WBH samples were evaluated by two different methods^30, 38^.

### Tissue distribution of Vitellogenin in male guppy exposed to E_2_

Adult male guppies (n = 16) were exposed to nominal concentrations of 50, 100, and 200 ng/L E_2_ in 5-L aquaria. The stock solution of E_2_ was prepared in ethanol and kept at 4 °C. The ethanol concentration in each group was below 0.001%. The exposure solution was renewed daily. After 21 days of exposure, fish were anesthetized in a bath of tricane methane sulfate, body lengths and weights were measured. The liver, eyes, testis, brain, and skin of each fish were separated with sterilized scissors and tweezers. Stainless steel blades were used to cut one third of caudal fins with an approximate weight of 0.02 g. Each tissue was weighed, diluted 1:4 (v/v) in 10 mM PBS containing 0.02% aprotinin, and homogenized by an automatic grinding machine (ShangHai Jingxin industrial development CO., LTD) with a homogeneous velocity of 50 HZ for 2 min. After centrifugation (8000 *g*, 10 min) at 4 °C, the supernatant was transferred to a clean tube for Vtg detection by Western blot and sandwich ELISA assay. All assays were carried out in duplicate.

### Vitellogenin induction in caudal fin of male guppy exposed to EE_2_ and BPS

Adult male guppies (n = 20) were exposed to 2, 10, and 50 ng/L EE_2_ and 1, 10, 100 μg/L BPS, respectively. After 21 days, caudal fin, liver tissue, and whole body were homogenated and centrifugated as described above. Vtg in the supernatant were quantified by the developed sandwich ELISA.

### Statistics

Vtg induction data are presented as the mean ± standard deviation, and the differences between the control and exposed groups were assessed by one-way analysis of variance followed by a Tukey’s post hoc tests. Prior to parametric analysis, data were log-transformed to achieve variance homogeneity. All statistical tests were conducted using SPSS 18.0 software (SPSS Inc., USA), and values were determined as significant when *P* < 0.05.

### Data Availability

All data has been included in the manuscript. The primary data is available to all interested researchers by contacting wangjun@ouc.edu.cn.

## Electronic supplementary material

Supplementary information
