# Supplementary material for: Vitellogenin induction in caudal fin of guppy (Poecilia reticulata) as a less invasive and sensitive biomarker for environmental estrogens
Source: Sci Rep. 2017 Aug 9;7:7647. doi: 10.1038/s41598-017-06670-6 (PMC5550507; doi:10.1038/s41598-017-06670-6)
Supplement: Supplementary file 1 — Supplementary information [file 41598_2017_6670_MOESM1_ESM.docx]

**Supporting Information**

**Vitellogenin induction** **in** **caudal fin of** **guppy (*Poecilia reticulata*) as a** **less invasive and sensitive** **biomarker for** **environmental estrogens**

Jun Wang, Shuwei Ma, Zhenzhong Zhang, Mingyi Zheng, Yifei Dong, Shaoguo Ru

Marine Life Science College, Ocean University of China, Qingdao 266003, China

This supporting information provides figures addressing (1) the matrix effects of caudal fin and whole-body homogenate samples in the routine ELISA assay; (2) how to collect caudal fin sample from male guppy for Vtg detection.

***Matrix effect***

Matrix effect is one of the most common challenges in performing immunoassays on complex samples. Non-Vtg compounds present in samples may affect the binding between the antibody and Vtg, thereby reducing the sensitivity and reliability of the immunoassay and possibly leading to false positive result. The common way to avoid such interferences is to dilute the sample with buffer. Therefore, the minimum dilution of tissue where matrix effect of the homogenate is negligible should be determined. We evaluated the matrix effect of caudal fin and whole-body homogenate samples by two different approaches described by Nilsen et al. (2004) and Holbech et al. (2001). The results obtained by the method of Nilsen et al. (2004) showed that the matrix effects for caudal fin and whole-body homogenate samples were found to be reduced to acceptable levels when they were diluted 20-fold and 40-fold, respectively. At these levels of dilution, the matrix interferences were somewhat similar to those observed in the case of the matrix free buffer (PBST) (Fig. S1).


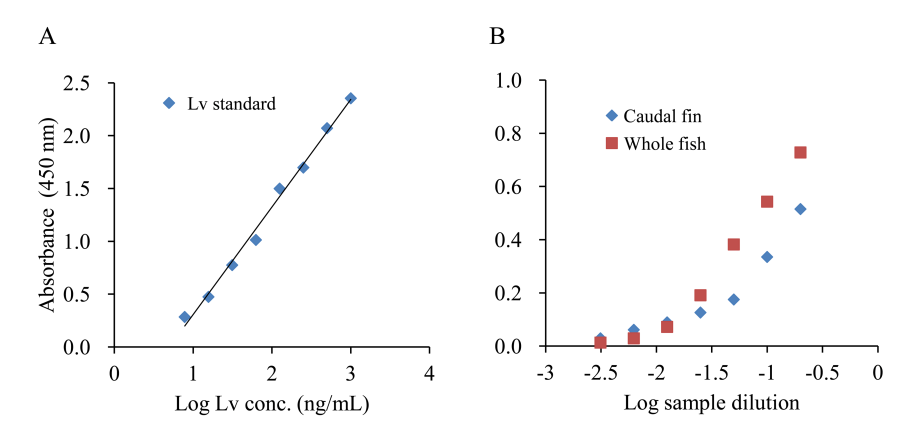


Fig. 1. Analysis of matrix effects in the developed sandwich ELISA. Dilution curves (twofold, diluted at 1:5, 1:10, 1:20, 1:40, 1:80; 1:160, and 1:320) of caudal fin and whole body homogenates (B) were compared with Lv standard curve (A). Samples from control males were used.

The matrix effects were also tested by adding the standard curve to a range of dilutions of caudal fin and whole body homogenates from control males (Holbech et al., 2001). The results showed that no clear matrix effects were observed for caudal fin homogenate diluted 1:20 and whole body homogenate diluted 1:40 (Fig. S2). Caudal fin and whole body homogenates were thus diluted at least 1:20 and 1:40 to avoid matrix effects during routine analyses. Based on the above experiments, the practical detection limit for Vtg in caudal fin and whole-body homogenate samples were thus 62 and 124 ng/mL, respectively.


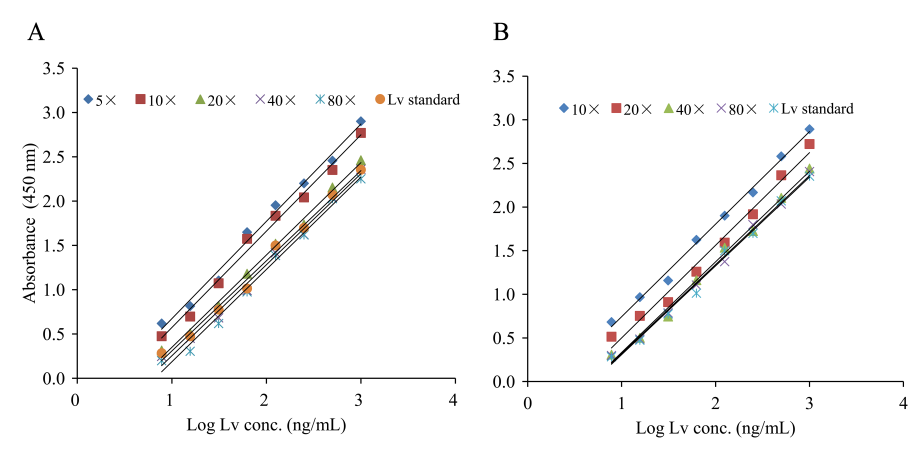


Fig. S2. Test of matrix effect. Caudal fin and whole body homogenate from male guppy were diluted 5, 10, 20, 40, 80 times and added standard curves ranging from 7.8 to1000 ng/mL.

***Caudal fin collection***

In these experiments, approximate one third of caudal fins with an average weight of 0.021±0.03 g in male guppy were cut from the posterior end (Fig. S3). To help the readers clearly understand the process of caudal fin sample collection, we have added this picture with a line drawn to indicate the cut position. Moreover, our study confirmed that the remove of one third of caudal fin would not cause the death of fish.


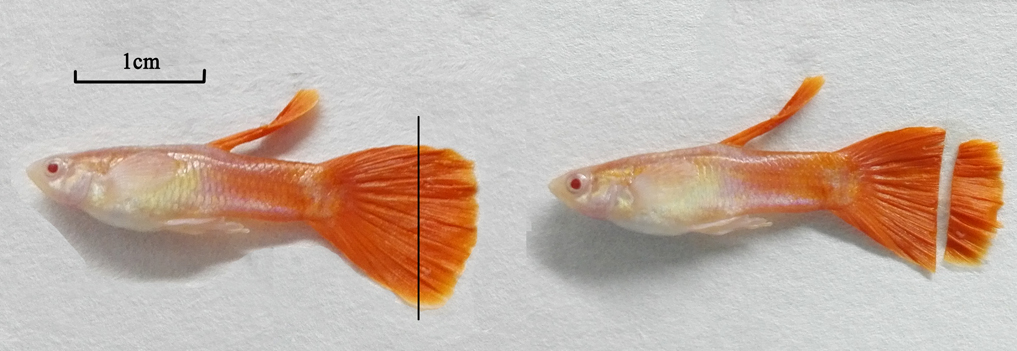


Fig. S3. The collection of caudal fin sample from male guppy for Vtg detection. The line indicated the position to cut caudal fin. Approximately one third oof caudal fin with an average weight of 0.021±0.003 g were removed.
